# Supplementary material for: Ustilago maydis PR-1-like protein has evolved two distinct domains for dual virulence activities
Source: Nat Commun. 2023 Sep 16;14:5755. doi: 10.1038/s41467-023-41459-4 (PMC10505147; doi:10.1038/s41467-023-41459-4)

Supplementary Information

Ustilago maydis PR-1-like protein has evolved two distinct domains for dual virulence activities

Yu-Han Lin[1#], Meng-Yun Xu[1#], Chuan-Chih Hsu[#1], Florensia Ariani Damei[1], Hui-Chun Lee[1], Wei-Lun Tsai[1], Cuong V. Hoang[1], Yin-Ru Chiang[2], and Lay-Sun Ma[1*]

[1]*Institute of Plant and Microbial Biology, Academia Sinica, Taipei 115201, Taiwan*

[2]*Biodiversity Research Center, Academia Sinica, Taipei 115201, Taiwan*

# These authors contributed equally.

Corresponding author:

Lay-Sun Ma (laysunma@gate.sinica.edu.tw)

This PDF file includes:

Supplementary Figure 1 to 6 and figure legends

Supplementary Table 1 to 3: contains information for strains, plasmids and primers.

Uncropped gels/dots/images of Supplementary figures

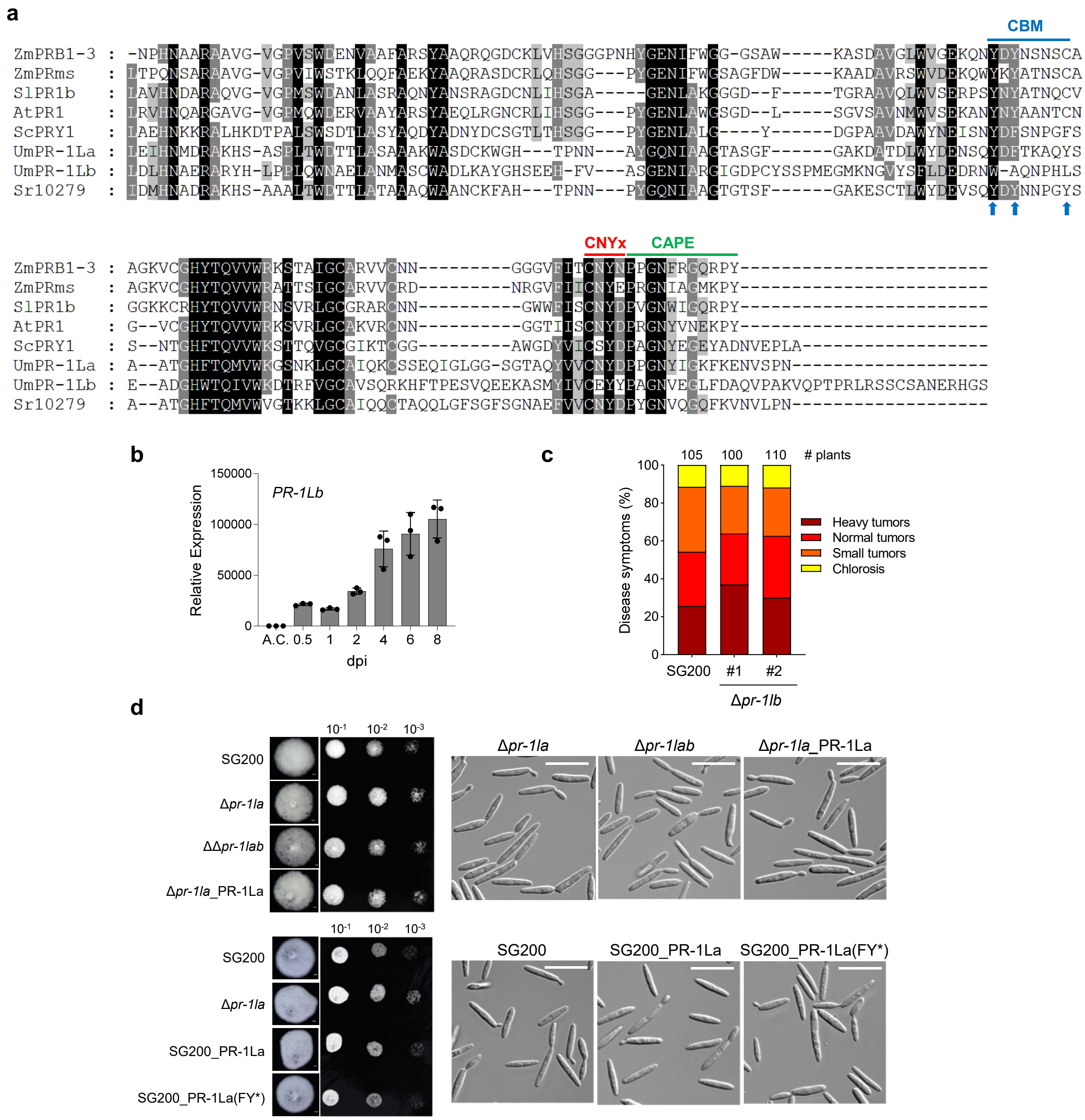

Supplementary Fig. 1: Analysis of CAP-domain sequences in PR-1 and PR-1L proteins, *UmPR-1Lb* gene expression, and virulence and cell morphology of SG200 variant strains. a In the sequence alignment of CAP domains, the plant CAPE peptides are depicted as a green line, while the caveolin binding motif (CBM) is indicated by a blue line. The conserved CNYx motif is depicted by a red line, and blue arrows denote the presence of aromatic amino acids in the CBM. *U. maydis* UmPR-1La (UMAG_01204; XP011387141); UmPR-1Lb (UMAG_04343; XP011391053); *Sporisorium reilianum* Sr10279 (CBQ70610); *Saccharomyces cerevisiae* ScPRY1 (NP012456); maize ZmPRB1-3 (DAA59477) and ZmPRms (NP001140745); *Solanum lycopersicum* SlPR1b (P04284); *Arabidopsis thaliana* AtPR1 (AEC06314).

Supplementary Fig. 1.

b qRT-PCR analysis of *PR-1Lb* gene expression in the SG200 strain, both during axenic culture (AC) and at various time intervals throughout the course of plant infection. Total RNA was extracted from SG200-infected maize leaves collected at the indicated days post-infection (dpi), and from cells grown in a liquid medium. The *U. maydis Peptidylprolyl Isomerase* (*PPI*) gene was used for normalization. The expression level of *PR-1Lb* in AC was set to 1.0. Average values of three biological replicates are shown. Error bars indicate ± standard deviation (SD). **c** Maize seedlings were infected with SG200 and two independent clones of the Δ*pr-1lb* mutant. Disease symptoms were scored at 12 dpi. Total numbers of infected plants from three independent infections are indicated above the respective columns. **d** The morphology of filamentous colonies and sporidial cells from the indicated strains was observed. These strains were either grown on PD-charcoal agar plates after serial dilution or cultured in a liquid medium. *PR-1La* expression in Δ*pr-1la* was controlled by the native promoter, while *PR-1La* and *PR-1La(FY*)* in SG200 were controlled by the *otef* promoter. Similar results were observed in at least two independent biological replicates. Bars, 20 μm. Source data are provided as a Source Data file.

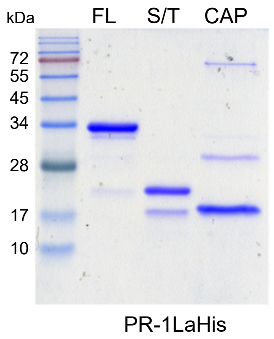

Supplementary Fig. 2: The purity analysis of recombinant C-terminal His-tagged PR-1La proteins. The indicated 6xHis-tagged recombinant proteins were expressed in *E. coli* cells without signal peptide. FL: full-length protein (24-279 amino acids.); S/T: Ser/Thr region (from 24 to 153 amino acids); CAP: CAP domain (from 126 to 279 amino acids). Similar protein purities were observed in three independent purification batches.

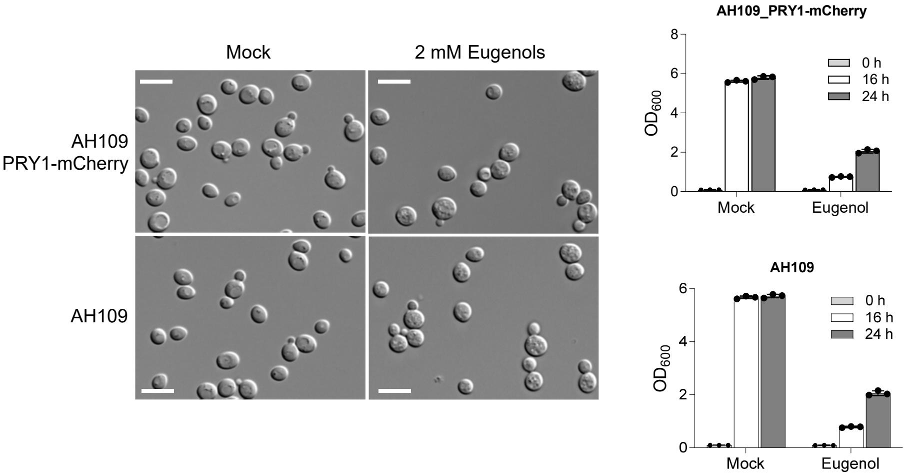

Supplementary Fig. 3: Yeast AH109 cells survive but do not induce pseudohyphae in the presence of eugenols. PRY1-mCherry fusion proteins were expressed in AH109 cells under the control of the ADH1 promoter. The indicated yeast cells, with an OD of approximately 0.1, were grown in YPD liquid medium supplemented with 2 mM of eugenols or 1.5% ethanol (mock) for 24 hours. Cell OD_{600} values were measured at the indicated time points. Values represent mean ± sd from three independent biological replicates. Images were taken after the 24-hour incubation. Similar cell morphologies were observed in at least two independent biological replicates. Bars, 10 μm. Source data are provided as a Source Data file.

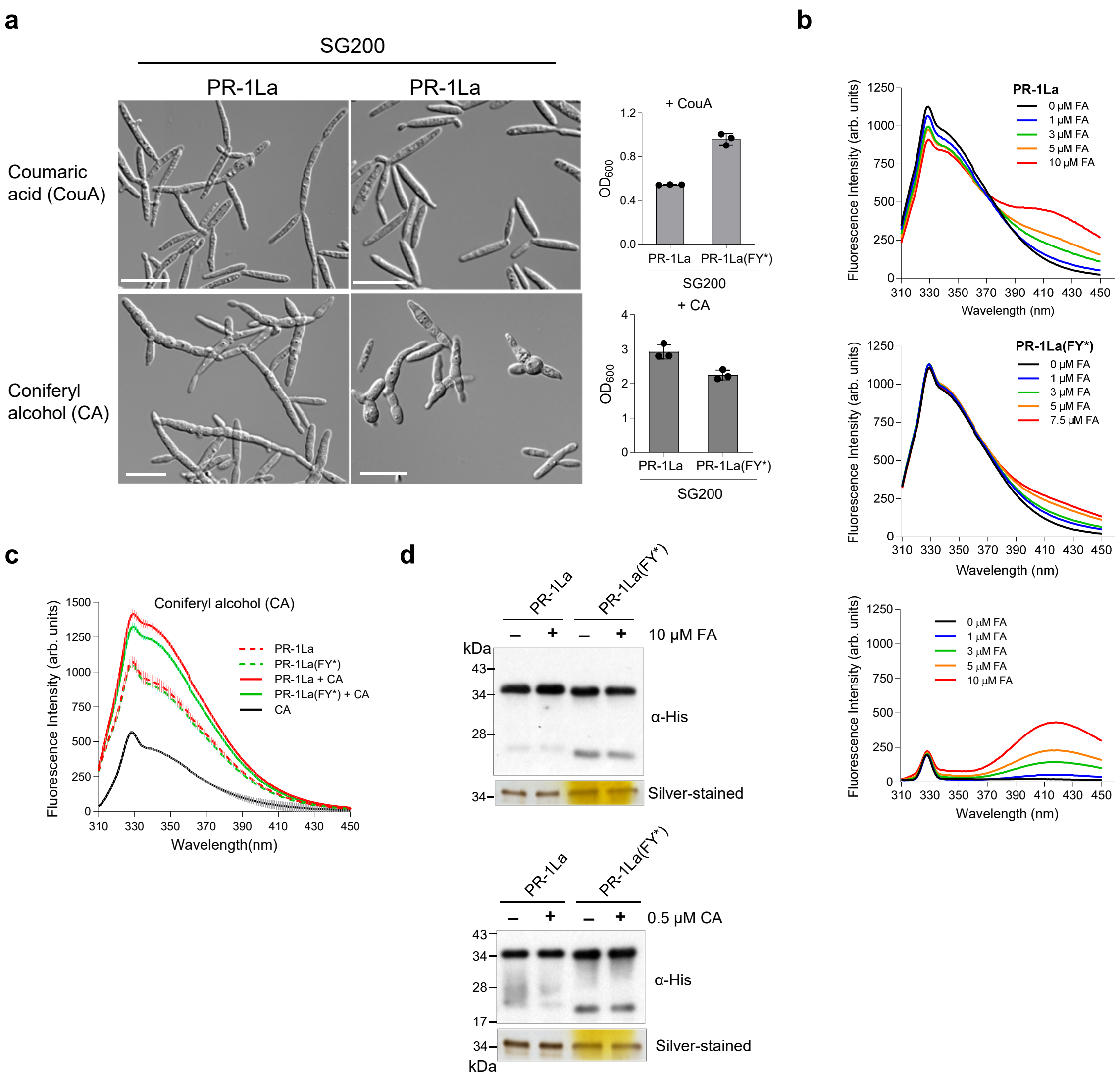

Supplementary Fig. 4: PR-1La binds to structural-related phenolics and induces hyphal-like structure.

a SG200 cells expressing indicated C-terminal HA-tagged proteins under the control of *otef* promoter were grown in YEPSL medium containing 5 mM of either coumaric acid (CouA) or coniferyl alcohol (CA) for 24 hours. Cell morphology was examined and the OD_{600} measurement was taken after the 24-hour incubation. Values indicate mean ± sd from three independent biological replicates showing similar cell morphologies. Bars, 20 µm. **b** Titration of recombinant proteins PR-1La and PR-1La (FY*) binding to ferulic acids. The intensities of fluorescence (in arbitrary units) were measured after incubating varying concentrations of ferulic acids (FA) with 0.25 µM of either PR-1La or PR-1La(FY*) in a 50 mM sodium acetate buffer (pH 5.5) at room temperature for 15 minutes. Values represent the mean from two independent measurements using two separate preparations of purified proteins.

Supplementary Fig. 4.

b, c Tryptophan residues in PR-1La proteins were selectively excited at 290 nm, and the emission spectra were recorded within the range of 310-450 nm. **c** The binding of PR-1La to coniferyl alcohol (CA). The indicated proteins (0.25 μM) was incubated with 0.5 μM CA in the sodium acetate buffer for 15 minutes before the fluorescent measurement. Values represent the mean from three independent measurements using two separate preparations of purified proteins. **d** The stability of recombinant proteins after exposure to the indicated phenolics (+) or buffer (-) at room temperature for 15 minutes. Protein samples were analyzed by immunoblotting using an anti-His antibody. One of three independent biological replicates with similar results is shown. Source data are provided as a Source Data file.

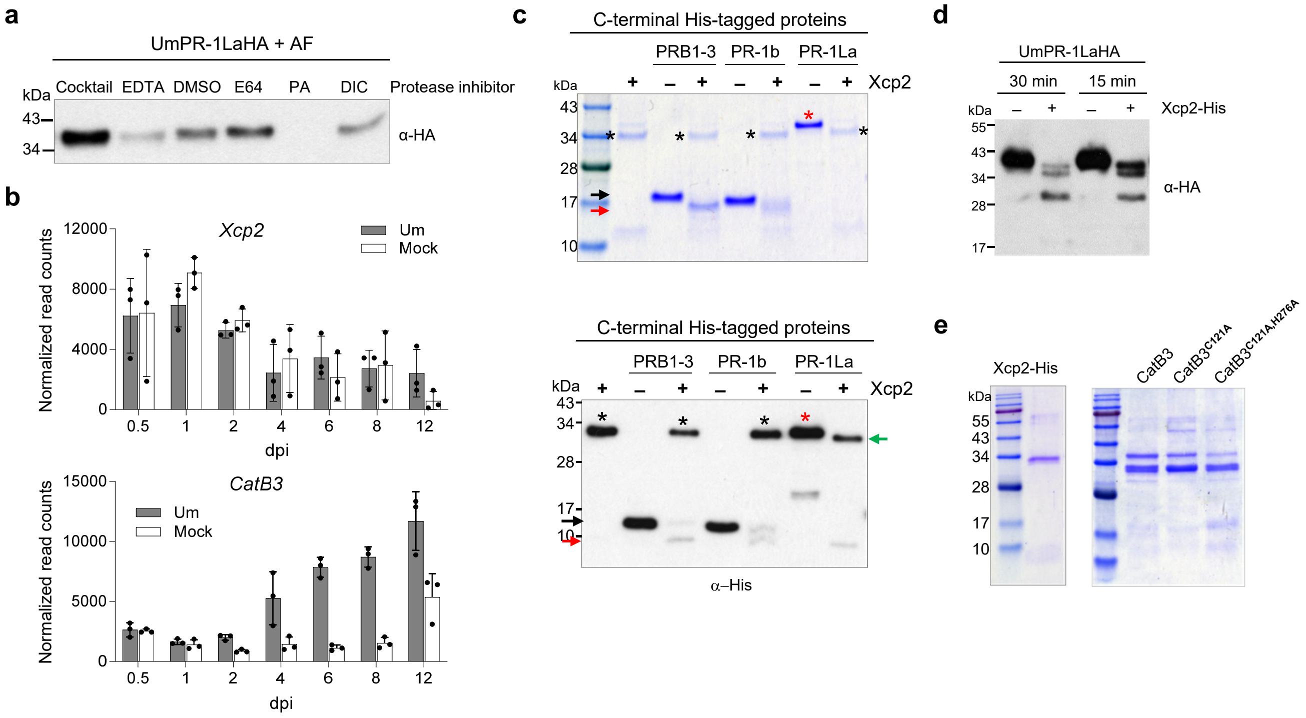

Supplementary Fig. 5: The cleavage analysis of plant PR-1 and PR-1La proteins by Xcp2 and CatB3. a Cleavage analysis of *U. maydis*-secreted PR-1LaHA (UmPR-1LaHA) in plant apoplastic fluid (AF). The culture supernatant of SG200_PR-1La cells was incubated with 30 µg of AF proteins from the SA-inoculated maize leaves, along with various protease inhibitors or 1 µl of DMSO (control). The reaction added either 0.1 mM protease inhibitor or 1 mM EDTA and was incubated at 28 °C for 2 hours. The protease inhibitor cocktail and E64 were dissolved in DMSO, which served as the negative control. DIC (3,4-Dichloroisocoumarin) and PA (Pepstatin A). One of two replicates showing similar results is shown. **b** Expression profiles of maize *Xcp2* (GRMZM2G066326; NP_001149806.1) and *CatB3* (GRMZM2G108849; ONM57676.1) at different time points of plant infection by *U. maydis* (Um) or H_2O control (mock). Data retrieved from an RNA-seq analysis[6]. dpi: days post infection. Values indicate mean ± sd from three biological replicates. **c** Cleavage of PR-1 and PR-1La proteins by Xcp2. All proteins contained C-terminal His-tags. After incubating PR-1 or PR-1La proteins with Xcp2 for 30 min in a sodium acetate buffer (pH 5.5), proteins were separated on SDS-PAGE, either stained by Coomassie-blue or analyzed by immunoblotting. Black and red asterisks (*) indicate full-length (FL) protein bands of Xcp2 and PR-1La, respectively. Black and red arrows indicate FL and truncated plant PR-1-His, respectively. Green arrow denotes the fragment is likely derived from PR-1La. **d** The cleavage of UmPR-1La (HA-tagged) by Xcp2. The concentrated culture supernatant of SG200_PR-1La cells was treated with Xcp2 proteins for indicated time points in a sodium acetate buffer, followed by immunoblotting analysis. One of the three independent replicates showing similar results is shown (**c, d**). **e** Purity of Xcp2-His, and CatB3 and its catalytically inactive variants (Cys121 and His276 were substituted with alanine). These proteases, containing the native signal peptides and no tags, were expressed and purified in parallel from the apoplasts of *N. benthamiana* leaves. Similar results were observed in at least two independent biological replicates. Source data are provided as a Source Data file.

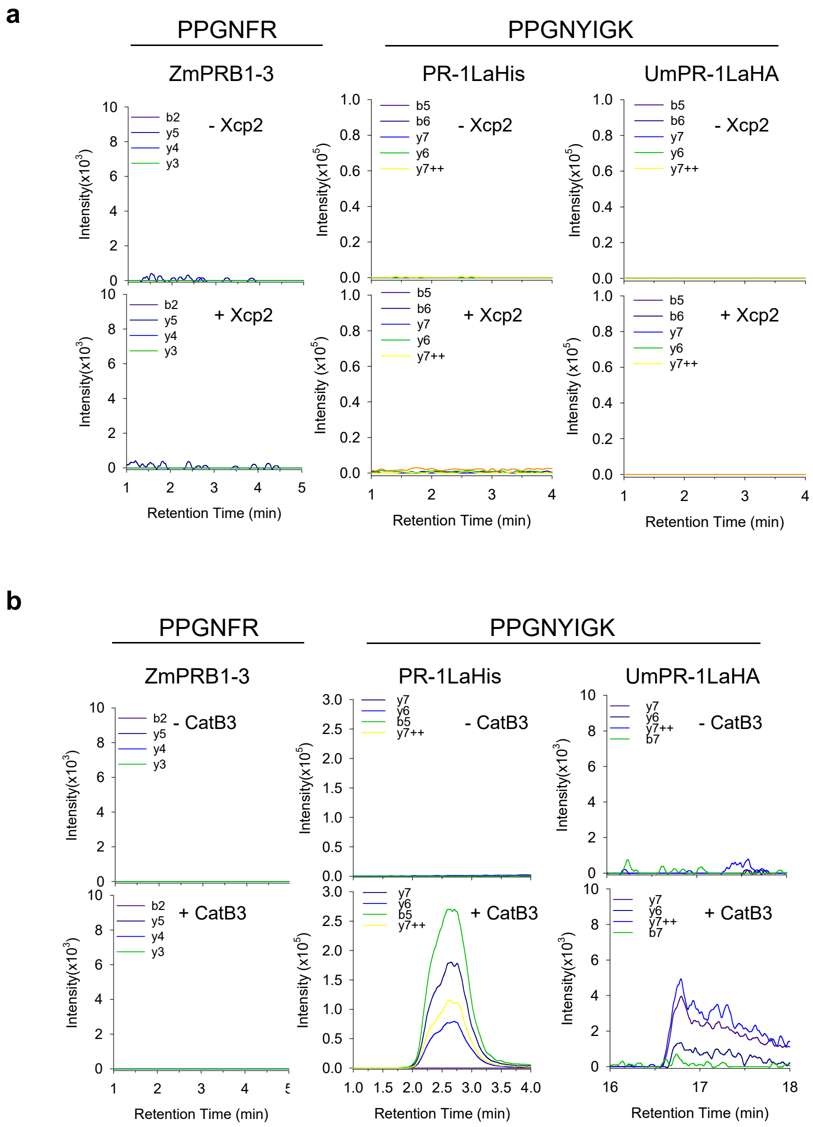

Supplementary Fig. 6: Detection of UmCAPE-La and ZmCAPE peptide by targeted LC-MS/MS analysis.

a, b Maize PRB1-3His, recombinant PR-1LaHis, or the culture supernatant fraction of SG200_PR-1La strain were incubated with Xcp2 (a) or CatB3 (b), followed by trypsin-digestion before targeted LC-MS/MS analysis. Expected short tryptic peptide sequences PPGNFR and PPGNYIGK derived from ZmCAPE and UmCAPE-La, respectively are shown. The quantity of the tryptic short peptide PPGNYIGK was measured in both the -CatB3 and +CatB3 samples using the peak area of fragment ions at specific retention times. Fragment ion b5 extends from the N-terminus, and y6 and y7 ions extend from the C-terminus, while y7++ is a doubly charged ion. The term "intensity" refers to the amplitude of the free induction decay signal. Two independent replicates using His- and HA-tagged PR-1La proteins are shown. Source data are provided as a Source Data file.

Supplementary Table 1 Strains used in this study

Strain	Genotype	Resistance*	Reference
SG200	*a1 mfa2 bW2 bE1, ble;*	P	[1]
AB33	*a2 Pnar: bW2 bE1*	P	[2]
AB33_ P_{otef}-*PR-1LaHA*	*a2 Pnar: bW2 bE1;ip*^{R} [P_{otef}-*PR-1LaHA] ip*^{S}	P, C	This work
SG200Δ*pr-1la*	*a1 mfa2 bW2 bE1, ble; umag01204(pr-1la)::nat*	P, N	This work
SG200Δ*pr-1lb*	*a1 mfa2 bW2 bE1, ble; umag04343(pr-1lb)::hyg*	P, H	This work
SG200ΔΔ*pr-1lab*	*a1 mfa2 bW2 bE1, ble; umag01204::nat;um04343::hyg*	P, C, N, H	This work
SG200Δpr-1la_P_{1204}-*PR-1La*	*a1 mfa2 bW2 bE1, ble; umag01204::nat; ip*^{R} [P_{1204}-*PR-1La] ip*^{S}	P, C, N	This work
SG200Δpr-1la_P_{1204}-*PR-1LaHA (FY*)*	*a1 mfa2 bW2 bE1, ble;umag01204::nat; ip*^{R} [P_{1204}-*pr-1la (FY*)] ip*^{S}	P, C, N	This work
SG200Δpr-1la_ P_{otef}-*PR-1LaHA*	*a1 mfa2 bW2 bE1, ble;umag01204::nat; ip*^{R} [P_{otef}-*PR-1LaHA] ip*^{S}	P, C, N	This work
SG200_ P_{otef}-*PR-1LbHA*	*a1 mfa2 bW2 bE1, ble;ip*^{R} [P_{otef}-*PR-1LbHA] ip*^{S}	P, C	This work
SG200_P_{otef}-*PR-1LaHA (FY*)*	*a1 mfa2 bW2 bE1, ble;ip*^{R}[P_{otef}-*PR-1LaHA (FY*)] ip*^{S}	P, C	This work
SG200_P_{otef}-*PRY1HA*	*a1 mfa2 bW2 bE1, ble; ip*^{R} [P_{otef}-*PRY1HA] ip*^{S}	P, C	This work
SG200_P_{otef}-*S/T*$_{PR-1La}$-*CAP*$_{PRY1}$*HA*	*a1 mfa2 bW2 bE1, ble; ip*^{R} [P_{otef}- *S/T*$_{PR-1La}$-*CAP*$_{PRY1}$*HA] ip*^{S}	P, C	This work
SG200_P_{otef}- *SP*$_{1204}$-*Sr10279HA*	*a1 mfa2 bW2 bE1, ble; ip*^{R} [P_{otef}- *SP*$_{1204}$-*Sr10279HA] ip*^{S}	P, C	This work
SG200_P_{1204}-*SP*$_{1204}$-*Sr10279*	*a1 mfa2 bW2 bE1, ble; ip*^{R} [P_{1204}-*SP*$_{1204}$-*Sr10279] ip*^{S}	P, C	This work
SG200Δpr-1la_P_{1204}-*PR-1La(CAPE-Lb)*	*a1 mfa2 bW2 bE1, ble; umag01204::nat; ip*^{R} [P_{1204}-*PR-1La(CAPE-Lb)] ip*^{S}	P, C, N	This work
SG200Δpr-1la_P_{1204}-*PR-1La(ZmCAPE)*	*a1 mfa2 bW2 bE1, ble; umag01204::nat; ip*^{R} [P_{1204}-*PR-1La(ZmCAPE)] ip*^{S}	P, C, N	This work

* *ble*: Phleomycin (P)-resistance gene; *nat*: *Streptomyces noursei* nourseothricin (N)-resistance-encoding gene; Hygromycin (H); Carboxin (C);

Supplementary Table 2 Plasmids used in this study

Plasmid name	Description	References
pGADT7	Yeast expression vector that is designed to express a protein of interest fused to a GAL4 activation domain	Clontech
p123	Plasmid containing the *GFP* gene controlled by constitutive promoter *otef, nos* terminator, the *U. maydis* carboxin resistant *ip* allele (ip^R), and ampicillin resistance gene. This plasmid served as backbone to insert gene of interest ectopically into the *U. maydis ip* locus.	[3]
pGADT7_PRY1-mCherry	Step1:<br>HindIII/XbaI-digested PCR fragment amplified from yeast AH109 gDNA using primer pairs #2/1 and XbaI/NotI-digested mCherry fragment were ligated into the HindIII/NotI-digested p123 plasmid to generate plasmid p123-pry1-mcherry.<br>Step2: NotI/HindIII-digested PCR fragment amplified from pGADT7 plasmid using primer pairs #3/4 and HindIII/NotI digested p*123-PRY1-mCherry* plasmid were combined and ligated into the HindIII-digested pGADT7.	This study
pAFP1-His	Plasmid containing maize *AFP1* gene used for producing the C-terminal His-tagged AFP1 protein in *Nicotiana benthamiana*.	[4]
pXcp2-His (maize)	A PCR fragment amplified from cDNA of maize B73 using primer pair #5/6 was integrated into the BsaI-digested p*AFP1-His* plasmid via Gibson assembly.	This study
pCatB3-His (maize)	A BsaI-digested PCR fragment amplified from cDNA of maize B73 using primer pair #7/8 was integrated into the BsaI-digested pAFP1-His plasmid via Gibson assembly.	This study
pCatB3-His (C121A; maize)	Two PCR fragments amplified from p*CatB3-His* plasmid using primer pairs #37/38 and #39/40 were combined and integrated into the Kpn1/Hpa1-digested p*CatB3-His* via Gibson assembly.	This study
pCatB3-His (C121A, H276A; maize)	Three PCR fragments amplified from p*CatB3-His* plasmid using three primer pairs #37/38, #39/41, and #42/40 were combined and integrated into the Kpn1/Hpa1-digested p*CatB3-His* via Gibson assembly.	This study
pPRB1-3-His (maize)	Two PCR fragments amplified from p*Xcp2-His* plasmid using primer pairs #20/21 and #22/23, and one PCR fragment amplified from cDNA of maize B73 using primer pairs #25/24 were integrated into the KpnI/SpeI-digested *pXcp2-His* via Gibson assembly.	This study
pPR1b-His (tomato)	Two PCR fragments amplified from p*Xcp2*-His plasmid using primer pairs #20/21 and #22/23, and one PCR fragment amplified from tomato (money maker) cDNA using primer pairs #26/27. The 3 fragments were combined and integrated into the KpnI/SpeI-digested *pXcp2-His* plasmid via Gibson assembly.	This study
P_{otef}-VP1HA	p123-derived plasmid used in constitutively expressing secreted Vp1HA in *U. maydis*.	[5]
P_{1204}-PR-1La	A KpnI/NotI-digested PCR fragment amplified from *U.maydis* gDNA using primer pairs #34/35 was ligated into the KpnI/NotI-digested *p*123 vector.	This study
P_{1204}-PR-1La(ZmCAPE)	A PCR fragment amplified from plasmid *P_{1204}-PR-1La* using primer pairs #49/50 was integrated into the PmlI/BamHI-digested *P_{1204}-PR-1La* plasmid via Gibson assembly.	This study
P_{1204}-PR-1La(CAPE-Lb)	Two PCR fragments amplified from plasmid *P_{1204}-PR-1La* and *P_{otef}-PR-1LbHA* using primer pairs #49/51 and #52/53, both of them were integrated into the PmlI/NotI-digested plasmid *P_{1204}-PR-1La* via Gibson assembly.	This study
P_{1204}- PR-1La(FY*)	Two PCR fragments amplified from plasmid *P_{1204}-PR-1La* using primer pairs #36/15 and #16/12, both of them were integrated into the BstXI/NotI-digested plasmid *P_{1204}-PR-1La* via Gibson assembly.	This study

Supplementary Table 2 Plasmids used in this study

Plasmid name	Description	References
P_{otef}- PR-1LaHA	A XbaI/XmaI-digested PCR fragment amplified from SG200 gDNA using primer pair #32/33 was ligated into the XbaI/XmaI-digested plasmid *P_{otef}-VP1HA.*	This study
P_{otef}-S/T_{PR-1La}-CAP_{PRY1}HA	One PCR fragment amplified from P_{otef}- pr-1laHA plasmid using primer pairs #9/10, the other PCR fragment amplified from P_{otef}- pr-1laHA plasmid with primer pair #11/12, both fragments were integrated into the XbaI/XmaI-digested plasmid *P_{otef}-VP1HA* via Gibson assembly.	This study
P_{otef}- PR-1LaHA(FY*)	A XbaI/XmaI-digested PCR fragment amplified from *P_{1204}-PR-1La(FY*)* plasmid using primer pairs #32/33 was ligated into the XbaI/XmaI digested plasmid *P_{otef}-VP1HA.*	This study
P_{1204}- SP_{1204}-Sr10279	Two PCR fragments were amplified from *P_{1204}-PR-1La* and gDNA of *S. reilianum* using primer pairs #43/44 and #45/46 respectively. Both fragments were integrated into the BstXI/NotI-digested plasmid *P_{1204}-PR-1La* via Gibson assembly.	This study
P_{otef}- SP_{1204}-Sr10279HA	A BamHI/XbaI-digested PCR fragment amplified from *P_{1204}- SP_{1204}-Sr10279* using primer pair #47/48 was ligated into the BamHI/XbaI-digested *P_{otef}-PR-1LaHA* plasmid.	This study
P_{otef}- PR-1LbHA	A XbaI/XmaI digested PCR fragment amplified from SG200 gDNA using primer pairs #30/31 was ligated into the XbaI/XmaI digested plasmid P_{otef}-vp1HA.	This study
P_{otef}-SP_{1204}-PRY1HA	PCR fragment amplified from *P_{otef}- PR-1LaHA* plasmid using primer pairs #9/28, and PCR fragment amplified from Yeast gDNA with primer pair #29/1, were integrated into the XbaI/XmaI-digested *P_{otef}- PR-1LaHA* plasmid via Gibson assembly.	This study
pET28a_PR-1La-His	NcoI/NotI digested PCR fragment amplified from plasmid *P_{1204}-PR-1La* using primer pair #13/14 was ligated into the NcoI/NotI-digested pET28a plasmid.	This study
pET28a_ PR-1La(FY*)-His	Two PCR fragments amplified from *pET28a_PR-1La-His* plasmid using primer pair #13/15 and #16/17, were integrated into the NcoI/NotI digested p*ET28a_PR-1La-His* plasmid via Gibson assembly.	This study
pET28a_ PR-1La(S/T)-His	NcoI/NotI digested PCR fragment amplified from pET28a_pr-1la-His plasmid using primer pair #13/18 was ligated into the NcoI/NotI-digested p*ET28a_PR-1La-His* plasmid.	This study
pET28a_PR-1La (CAP)-His	NcoI/NotI digested PCR fragment amplified from pET28a_pr-1la-His plasmid using primer pair #19/14 was ligated into the NcoI/NotI digested p*ET28a_PR-1La-His* plasmid.	This study

Supplementary Table 3 Oligonucleotides used in this study

Oligonucleotides for plasmid construction	
**Name**	**Sequence (5' to 3')**
#1	GACGTCGTAGGGATATCTAGAAGCTAGGGGCTCGACATTATC
#2	CCAAGCTTTGCAAAGATGAAACTTTCTAAATTATCGATCTTAACCTCCGCCTTAGC
#3	GTTGTCGGCTTGTCTACCTT
#4	AAAGCGGCCGCTCGAGCTGCAGATGAATCGTAG
#5	TTGGTCTCAAGGTATGGCTTGGTCTTGTGCTCG
#6	TTGGTCTCAAAGCTCAATGGTGGTGGTGGTGATGGTCCTTGGTCGGGTAG
#7	TTGGTCTCAAGGTATGGGCGGCGCACTGCTGCTTG
#8	TTGGTCTCAAAGCTTAATGGTGGTGGTGGTGATGAACTATAGCTCTTCCAACG
#9	CTGTCTCGGCACTATCTTTC
#10	TCTGACAGATTCGAGTCGGCGGTTC
#11	CCGCCGACTCGAATCTGTCAGATTTTGCCTCTTC
#12	CATCGCAAGACCGGCAACAGGATTC
#13	ACTTTAAGAAGGAGATATACCATGGGATCTCAGCCTGTCATCGAGC
#14	TTAGCGGCCGCGTTGGGCGAGACGTTCTC
#15	GCTAGCCTGTGCCTTGGTGGCATCATACTGAGAGTTTTCATC
#16	GCCACCAAGGCACAGGCTAGCGCTGCCACGGGACATTTCAC
#17	TCAAGACCCGTTTAGAGG
#18	GTGCGGCCGCCGACGCTGAGTGTTTGGCTCGATCC
#19	ACGCCATGGGAACCGCCGACTCGAATCTG
#20	CCTGTGGTTGGCACATAC
#21	CCATACCTGCAACAATAAGAAC
#22	CATCACCACCACCACCAT
#23	AATGTACTGGGGTGGATG
#24	GTTCTTATTGTTGCAGGTATGGAGTACTCGTCTACTAG
#25	TTAATGGTGGTGGTGGTGATGGTAGGGTCTCTGTCCGCGGAAG
#26	GTTCTTATTGTTGCAGGTATGGGGTTGTTCAACATCTC
#27	TTAATGGTGGTGGTGGTGATGGTAAGGACGTTGTCCGATCC
#28	GCGCAAGAGACTGACGCAAG
#29	CTTGCGTCAGTCTCTTGCGCCGCTCCTGCCGTTGTTACTGTC
#30	TTACCCGGGATGGCGCTGGTCGCCATG

Supplementary Table 3 Oligonucleotides used in this study

Name	Sequence (5' to 3')
#31	GCTCTAGAACTGCCGTGTCTCTCGTTAG
#32	CCCCCGGGATGAAAGTCACATCTGTGATCG
#33	ATATCTAGAGTTGGCGAGACGTTCTC
#34	CTCGGTACCTTCTCATAACGAATTGTTC
#35	AATGCGGCCGCTCTAGTTGGCGAGACGTTC
#36	CGAGACTCATCGCTTGAGTG
#37	AGGCGGGAAACGACAATCTG
#38	GCCCATGCAGAGCCACAGTGACCTTGATCAAGTATGTTC
#39	TACTTGATCAAGGTCACTGTGGCTCTGCATGGGCTTTTGGTGCTGTGGA
#40	AAGACCGGCAACAGGATTCAATC
#41	ACGGCTGCGCCACCCATAATGCCACCGGTGATG
#42	CATCACCGGTGGCATTATGGGTGGCGCAGCCGTCAAGTTGATTGGATG
#43	ATGTTGCCCACGTCGAATCAAG
#44	CCCGCTCGACCGCGGGCTGAGAAGAGACTGACGCAAGGGCGGCAAA
#45	GTCAGTCTCTTCTCAGCCCGCGGTCGAGCGGGACAGCCTCGCCG
#46	TGAACGATCTGCAGCCGGGCGGCCGCTCAGTTGGGCAGAACATTCACC
#47	ACGGGATCCCCCGGGATGAAAGTCACATCTG
#48	ATATCTAGAGTTGGGCAGAACATTCACC
#49	CGGAAGCCTGCATTCCAAAC
#50	GGGCGGCCGCCTAGTAAGGACGTTGTCCGATATAGTTTCCAGGTG
#51	ACGTTGCCTGCCGGGTCATAGTTGCACACG
#52	TATGACCCGGCAGGCAACGTCGAAG
#53	CGATCTGCAGCCGGGCGGCCGCTCAACTGCCGTGTCTCTCG
**Oligonucleotides for qRT-PCR**	
**Name**	**Sequence (5' to 3')**
ZmPR5-F	TATCGGCCGGAATAGGCTCTG
ZmPR5-R	CGCGTACATACAAATGCGTGC
ZmPR1-F	TACAATGGAGGCATCCAACA
ZmPR1-R	CTGTTTTGGGGAGTGAGGTA
ZmPR2-F	CTCTTCTACGGCAACAAGCAGC
ZmPR2-R	AAGTCCGGCGGCCACATACAT
ZmGADPH-F	CTTCGGCATTGTTGAGGGTTTG
ZmGADPH-R	TCCTTGGCTGAGGGTCCGTC
PR-1La-F	TGCCACCGACTTGTGGTATG
PR-1La-R	GAGCACTTTTGAATCGCGCA
PR-1Lb-F	AATGCTGAGAAGTCGGGCAA
PR-1Lb-R	GCTGCTGGTTGGAAAATGGG

Supplementary References

1. Kamper, J. *et al.* Insights from the genome of the biotrophic fungal plant pathogen *Ustilago maydis*. *Nature* **444**, 97-101, doi:10.1038/nature05248 (2006).
2. Brachmann, A., Weinzierl, G., Kamper, J. & Kahmann, R. Identification of genes in the bW/bE regulatory cascade in *Ustilago maydis. Mol Microbiol* **42**, 1047-1063, doi:10.1046/j.1365-2958.2001.02699.x (2001).
3. Loubradou, G., Brachmann, A., Feldbrugge, M. & Kahmann, R. A homologue of the transcriptional repressor Ssn6p antagonizes cAMP signalling in *Ustilago maydis*. *Mol Microbiol* **40**, 719-730 (2001).
4. Ma, L. S. *et al.* The *Ustilago maydis* repetitive effector Rsp3 blocks the antifungal activity of mannose-binding maize proteins. *Nat Commun* **9**, 1711, doi:10.1038/s41467-018-04149-0 (2018).
5. Hoang, C. V., Bhaskar, C. K. & Ma, L. S. A Novel Core Effector Vp1 Promotes Fungal Colonization and Virulence of *Ustilago maydis*. *J Fungi (Basel)* **7**, doi:10.3390/jof7080589 (2021).
6. Lanver, D. *et al.* The Biotrophic Development of *Ustilago maydis* Studied by RNA-Seq Analysis. *Plant Cell* **30**, 300-323, doi:10.1105/tpc.17.00764 (2018).

Uncropped scans of gels/blots/images of Supplementary figures

Supplementary Fig. 1d

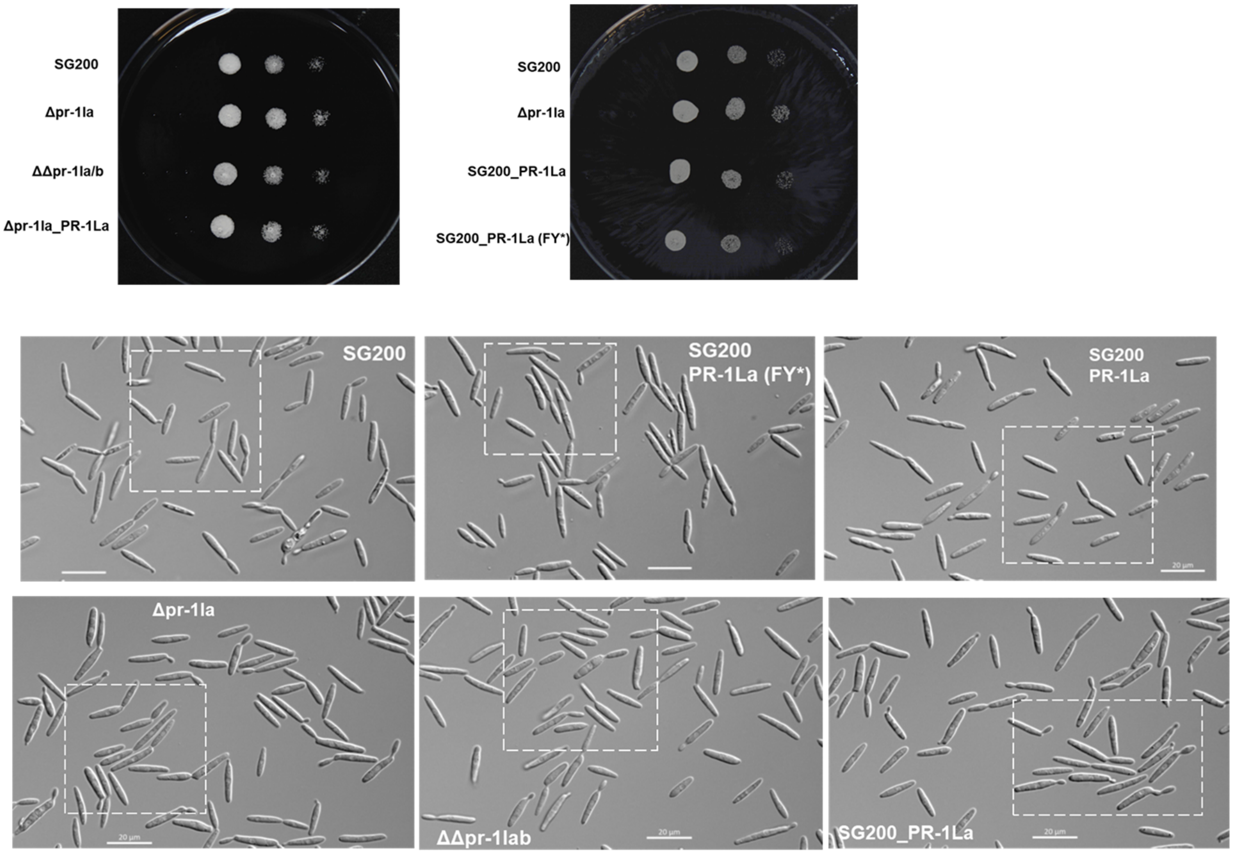

Supplementary Fig. 3

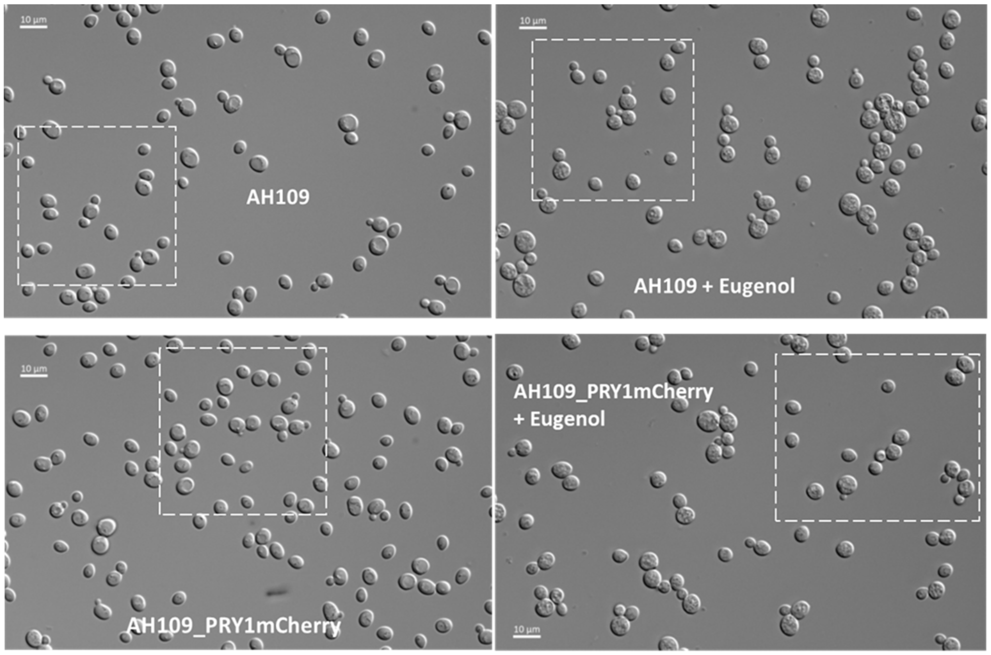

Supplementary Fig. 4a

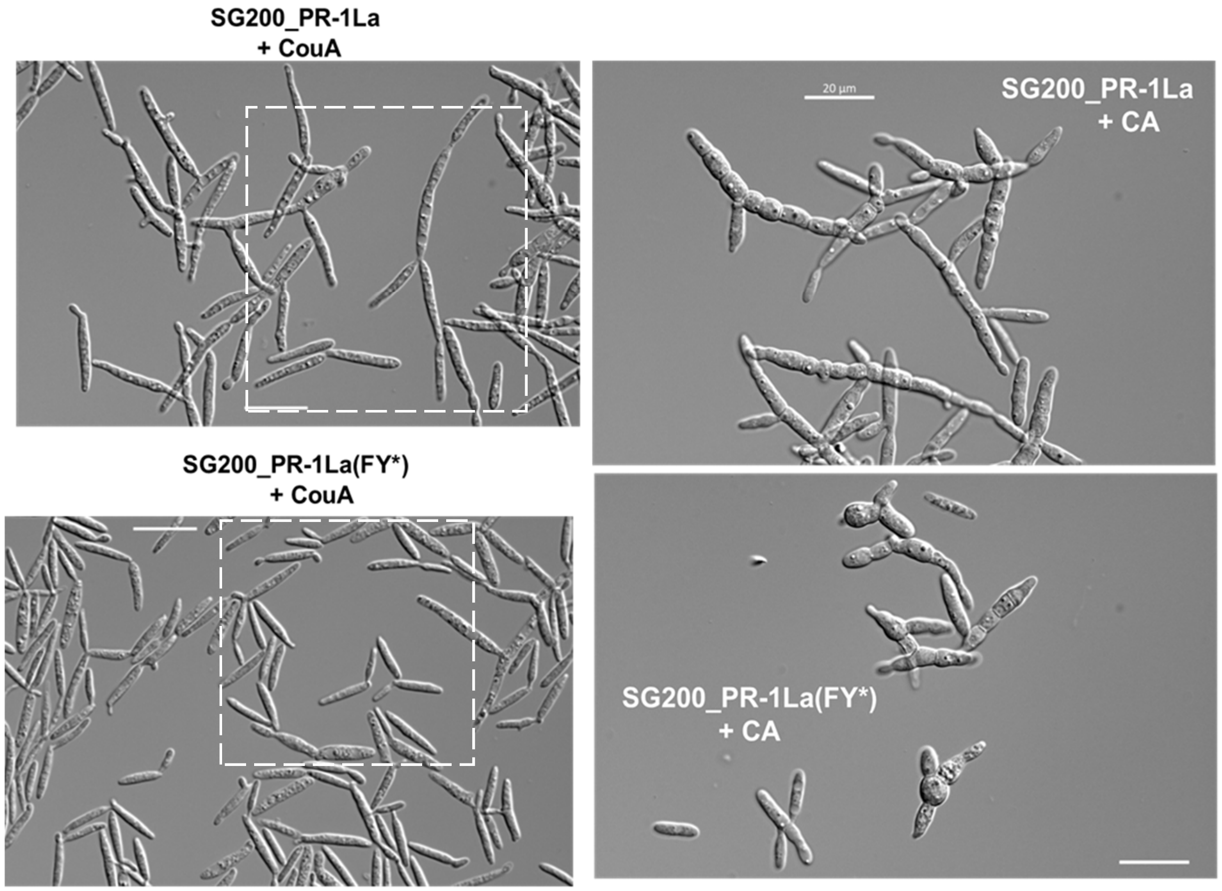

Supplementary Fig. 4d

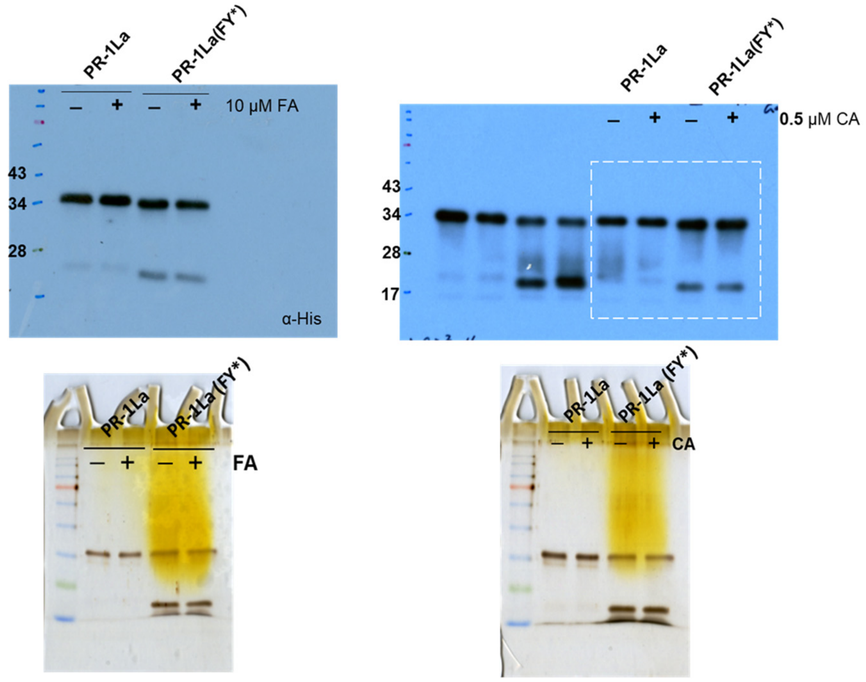

Supplementary Fig. 5a

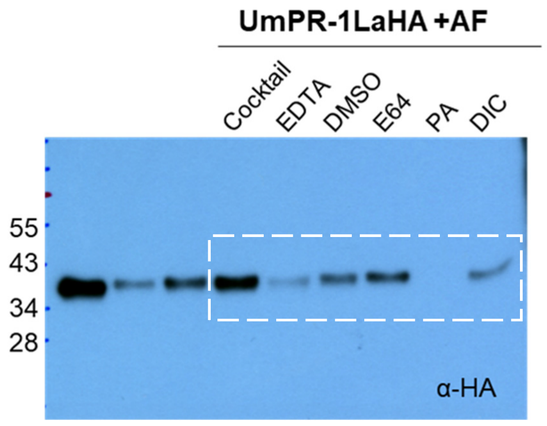

Supplementary Fig. 5c

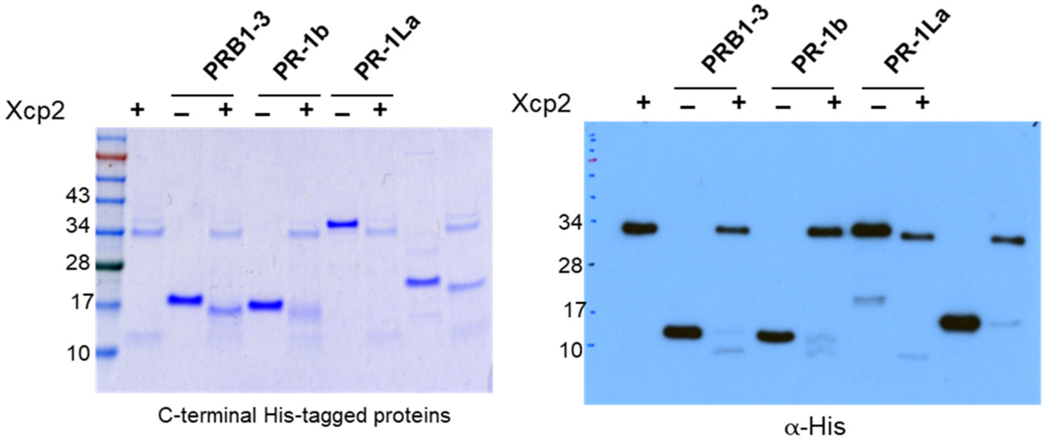

Supplementary Fig. 5d

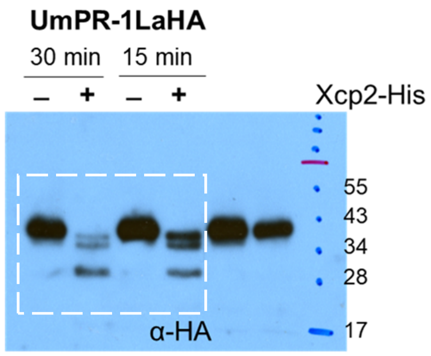

Supplementary Fig. 5e

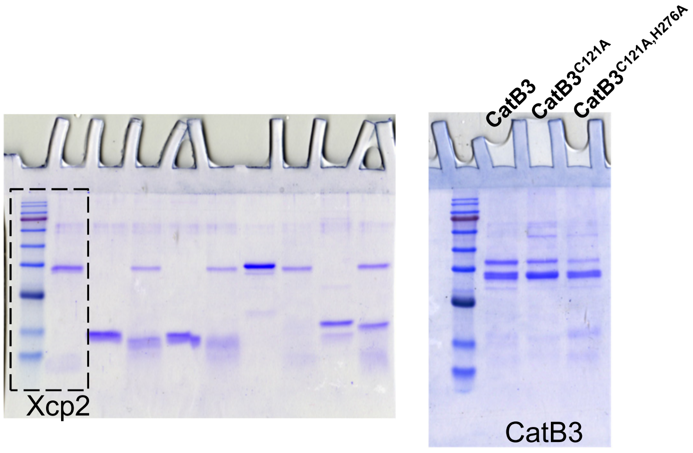

Supplement: Supplementary file 1 — Supplementary Information [file 41467_2023_41459_MOESM1_ESM.pdf]
