# Supplementary material for: Ustilago maydis PR-1-like protein has evolved two distinct domains for dual virulence activities
Source: Nat Commun. 2023 Sep 16;14:5755. doi: 10.1038/s41467-023-41459-4 (PMC10505147; doi:10.1038/s41467-023-41459-4)
Supplement: Supplementary file 3 — Reporting Summary [file 41467_2023_41459_MOESM3_ESM.pdf]

nature portfolio

Corresponding author(s): Lay-Sun Ma

Last updated by author(s): 2023/09/02

Reporting Summary

Nature Portfolio wishes to improve the reproducibility of the work that we publish. This form provides structure for consistency and transparency in reporting. For further information on Nature Portfolio policies, see our Editorial Policies and the Editorial Policy Checklist.

Statistics

For all statistical analyses, confirm that the following items are present in the figure legend, table legend, main text, or Methods section.

n/a	Confirmed	
☐	☒	The exact sample size (*n*) for each experimental group/condition, given as a discrete number and unit of measurement
☐	☒	A statement on whether measurements were taken from distinct samples or whether the same sample was measured repeatedly
☐	☒	The statistical test(s) used AND whether they are one- or two-sided<br>*Only common tests should be described solely by name; describe more complex techniques in the Methods section.*
☒	☐	A description of all covariates tested
☒	☐	A description of any assumptions or corrections, such as tests of normality and adjustment for multiple comparisons
☐	☒	A full description of the statistical parameters including central tendency (e.g. means) or other basic estimates (e.g. regression coefficient) AND variation (e.g. standard deviation) or associated estimates of uncertainty (e.g. confidence intervals)
☐	☒	For null hypothesis testing, the test statistic (e.g. *F*, *t*, *r*) with confidence intervals, effect sizes, degrees of freedom and *P* value noted<br>*Give P values as exact values whenever suitable.*
☒	☐	For Bayesian analysis, information on the choice of priors and Markov chain Monte Carlo settings
☒	☐	For hierarchical and complex designs, identification of the appropriate level for tests and full reporting of outcomes
☒	☐	Estimates of effect sizes (e.g. Cohen's *d*, Pearson's *r*), indicating how they were calculated

Our web collection on statistics for biologists contains articles on many of the points above.

Software and code

Policy information about availability of computer code

Data collection	To collect qRT-PCR data, we used the QuantStudio 12K Flex system (software version 1.2.2). For immunolocalization experiments, an Axio Observer fluorescence microscope equipped with an Axiocam 702 Monochrome camera (ZEISS, Germany) was employed, and the images were processed using ZEN 3.2 imaging software (ZEISS). Fluorescence intensity data was collected using a Jasco fluorescence spectrophotometer FP-8300 equipped with Spectra Manager software (version 2). The peptide analysis was conducted using Thermo Fisher Scientific Q-Exactive mass spectrometer coupled with an Ultimate 3000, controlled by Xcalibur Software (version 4.3.73.11). For the phylogenetic analysis, protein sequences collected from the NCBI database.
Data analysis	PRM raw files were analyzed using Skyline (version 22.2). Fragment spectra predictions for PPGNYIGK and PPGNFR were conducted using Prosit (https://www.proteomicsdb.org/prosit/). GraphPad Prism 10 was used for plotting graphs and conducting t-tests to analyze the data. Protein sequences collected from the NCBI database was aligned using ClustalOmega (https://www.ebi.ac.uk/Tools/msa/clustalo/). Alignment sequences were trimmed using trimAl (version 1.4.1). Evolutionary analysis was performed in MEGA 7.0, and the phylogenetic tree was generated using Maximum Likelihood method with the WAG+G+I model and 1000 bootstrap replicates. The consensus sequence of CAPE and CAPE-like peptides was identified using MEME (version 5.5.2). The scrambled peptide is randomly generated using Scrambled tool in Mimotopes website (http://www.mimotopes.com/peptideLibraryScreening.asp?id=97).

For manuscripts utilizing custom algorithms or software that are central to the research but not yet described in published literature, software must be made available to editors and reviewers. We strongly encourage code deposition in a community repository (e.g. GitHub). See the Nature Portfolio guidelines for submitting code & software for further information.

Data

Policy information about availability of data

All manuscripts must include a data availability statement. This statement should provide the following information, where applicable:

- Accession codes, unique identifiers, or web links for publicly available datasets
- A description of any restrictions on data availability
- For clinical datasets or third party data, please ensure that the statement adheres to our policy

Accession codes for protein sequences obtained from the NCBI database were provided in this study. Data relevant for CatB3 and Xcp2 gene expression analysis were extracted from the RNAseq dataset available at NCBI Gene Expression Omnibus under accession number GSE103876. The data that support the findings of this study are available within this manuscript and its Supplementary Information and Supplementary Data file. Source data are provided with this paper. MS raw data files for targeted MS analysis have been deposited to the ProteomeXchange Consortium via the PRIDE partner repository, under the dataset identifier PXD044915. All materials used in this study are described in the Supplementary Information. The unprocessed data, gels, and blots are provided in the Supplementary Information/Source Data file. Data or materials generated for this study is also available from the corresponding author upon request.

Research involving human participants, their data, or biological material

Policy information about studies with human participants or human data. See also policy information about sex, gender (identity/presentation), and sexual orientation and race, ethnicity and racism.

Reporting on sex and gender	n/a
Reporting on race, ethnicity, or other socially relevant groupings	n/a
Population characteristics	n/a
Recruitment	n/a
Ethics oversight	n/a

Note that full information on the approval of the study protocol must also be provided in the manuscript.

Field-specific reporting

Please select the one below that is the best fit for your research. If you are not sure, read the appropriate sections before making your selection.

☒ Life sciences ☐ Behavioural & social sciences ☐ Ecological, evolutionary & environmental sciences

For a reference copy of the document with all sections, see nature.com/documents/nr-reporting-summary-flat.pdf

Life sciences study design

All studies must disclose on these points even when the disclosure is negative.

Sample size	No sample size calculation was performed. For disease symptom analysis, we conducted at least three independent biological replicates, with each replicate consisting of a minimum sample size of 28 plants. This approach was chosen to ensure statistical relevance. For PR gene expression analysis, due to the varying response of seedlings, more than three replicates (each replicate with 8-10 plants) were performed to obtain large sample size to avoid the influence of random effects.
Data exclusions	Replicates were disregarded when the positive and/or negative controls failed to exhibit the anticipated results.
Replication	In this study, where representative pictures are shown, the experiments had been repeated at least twice, yielding similar results.
Randomization	Maize seedlings were used as the experimental organisms and were grown in a walk-in growth chamber under standardized conditions. Only 7-day-old seedlings with a similar size and at a stage with three developed leaves were used for the infections.
Blinding	Blinding did not apply to our study. The only individuals blinded to group allocation were the instrument operators during data collection for instrumental analysis.

Reporting for specific materials, systems and methods

We require information from authors about some types of materials, experimental systems and methods used in many studies. Here, indicate whether each material, system or method listed is relevant to your study. If you are not sure if a list item applies to your research, read the appropriate section before selecting a response.

Materials & experimental systems

n/a	Involved in the study
☐	☒ Antibodies
☒	☐ Eukaryotic cell lines
☒	☐ Palaeontology and archaeology
☒	☐ Animals and other organisms
☒	☐ Clinical data
☒	☐ Dual use research of concern
☐	☒ Plants

Methods

n/a	Involved in the study
☒	☐ ChIP-seq
☒	☐ Flow cytometry
☒	☐ MRI-based neuroimaging

Antibodies

Antibodies used	Mouse anti-HA monoclonal primary antibody (1:8,000 dilution, Yao-Hong Biotech., Taiwan; #YH80007)<br>Mouse anti-His monoclonal primary antibody (1: 8,000 dilution, Yao-Hong Biotech., Taiwan; #YH80003)<br>Mouse anti-α-Tubulin monoclonal primary antibody (1:8,000 dilution, Sigma, #T6199)<br>Goat anti-mouse IgG (H&L), HRP conjugated secondary antibody (1:25,000, Yao-Hong Biotech., Taiwan; #AS111772)<br>Goat-anti mouse IgG (H&L), Alexa Fluor 488-conjugated secondary antibody (1:2,000; Invitrogen #A28175)<br>Goat-anti mouse IgG (H&L), Alexa Fluor 594-conjugated secondary antibody (1:2,000; Abcam # AB150116)
Validation	All antibodies targeting epitope-tags were validated by either testing them on strains that did not express the epitope-tagged protein through western blotting/immunolocalization in this study or by referring to previous studies for validation (DOI: https://doi.org/10.1128/mbio.00093-23; https://doi.org/10.3390/jof7080589).
